# Amino Acid Intakes Are Associated With Bone Mineral Density and Prevalence of Low Bone Mass in Women: Evidence From Discordant Monozygotic Twins

**DOI:** 10.1002/jbmr.2703

**Published:** 2015-09-30

**Authors:** Amy Jennings, Alexander MacGregor, Tim Spector, Aedín Cassidy

**Affiliations:** ^1^ Department of NutritionNorwich Medical SchoolUniversity of East AngliaNorwichUK; ^2^ Department of Twin Research and Genetic EpidemiologyKings College LondonLondonUK

**Keywords:** PROTEIN, AMINO ACID, BONE MINERAL DENSITY, OSTEOPOROSIS, DIET

## Abstract

Although a higher protein intake, particularly from vegetable sources, has been shown to be associated with higher bone mineral density (BMD) the relative impact of specific amino acids on BMD and risk of osteoporosis remains to be determined. Mechanistic research suggests that a number of specific amino acids, including five nonessential amino acids—alanine, arginine, glutamic acid, glycine, and proline—may play a role in bone health, principally through improved production of insulin and insulin‐like growth factor 1 and the synthesis of collagen and muscle protein. However to date, no previous studies have examined the associations between habitual intake of amino acids and direct measures of BMD and prevalence of osteoporosis or osteopenia, and no studies have examined this relationship in discordant identical twin‐pairs. In these analyses of female monozygotic twin‐pairs discordant for amino acid intake (*n* = 135), twins with higher intakes of alanine and glycine had significantly higher BMD at the spine than their co‐twins with within‐pair differences in spine‐BMD of 0.012 g/cm^2^ (SE 0.01; *p* = 0.039) and 0.014 g/cm^2^ (SE 0.01; *p* = 0.026), respectively. Furthermore, in cross‐sectional multivariable analyses of 3160 females aged 18 to 79 years, a higher intake of total protein was significantly associated with higher DXA‐measured BMD at the spine (quartile Q4 to quartile Q1: 0.017 g/cm^2^, SE 0.01, *p* = 0.035) and forearm (Q4 to Q1: 0.010 g/cm^2^, SE 0.003, *p* = 0.002). Intake of six amino acids (alanine, arginine, glutamic acid, leucine, lysine, and proline) were associated with higher BMD at the spine and forearm with the strongest association observed for leucine (Q4 to Q1: 0.024 g/cm^2^, SE 0.01, *p* = 0.007). When intakes were stratified by protein source, vegetable or animal, prevalence of osteoporosis or osteopenia was 13% to 19% lower comparing extreme quartiles of vegetable intake for five amino acids (not glutamic acid or proline). These data provide evidence to suggest that intake of protein and several amino acids, including alanine and glycine, may be beneficial for bone health, independent of genetic background. © 2015 The Authors. *Journal of Bone and Mineral Research* Published by Wiley Periodicals, Inc. on behalf of the American Society for Bone and Mineral Research.

## Introduction

There is currently no clear consensus on the role of protein in bone health because protein may have competing effects on bone. Higher protein intake is thought to beneficially affect bone health through a number of mechanisms, including its role in maintaining bone structure and increasing insulin‐like growth factor 1 (IGF‐1), an important mediator of osteoblastic activity.^1^, ^2^ Conversely, dietary protein is a source of metabolic acid that may lower the pH of urine and increase urinary calcium excretion, which could lower bone mass,^3^ although a meta‐analyses of randomized trials reported that higher protein intakes are not detrimental to calcium retention or bone mineral loss.^4^ A systematic review on the relationship between protein and bone health reported no adverse associations between protein intake and bone mineral density (BMD) and a meta‐analysis of six randomized controlled trials showed a beneficial effect of protein supplementation (40 mg of milk basic protein or 20.4 g total protein) on lumbar spine BMD of 0.02 g/cm^2^.^5^ A number of cross‐sectional and prospective studies have also reported associations between higher total protein intake and higher BMD in women.^6^, ^7^, ^8^, ^9^ Data indicate that protein source (animal or vegetable) may influence the relationship between protein intake and bone health because protein from animal sources is high in acidic amino acids such as cysteine and methionine,^3^ although these acidic amino acids are also found in plant‐based sources such as nuts. Three recent studies have shown that vegetable protein, but not animal protein, is associated with increased BMD in women.^10^, ^11^, ^12^ Conversely, in a study of 562 women, increased intake of 15 g animal protein per day was associated with increased BMD at the hip (0.016 g/cm^2^) and femoral neck (0.012 g/cm^2^), and a 15 g/day increase in vegetable protein intake was associated with lower BMD at the hip (–0.013 g/cm^2^) and femoral neck (–0.010 g/cm^2^),^13^ and in a study of post‐menopausal women the relative risk of hip fracture was found to decrease significantly across increasing quartiles of animal protein intake (1.00 [reference]; 0.59 [95% CI, 0.3 to 1.3]; 0.63 [95% CI, 0.3 to 1.4]; and 0.31 [95% CI, 0.1 to 0.9]).^14^

To date, no human studies have looked at the potential impact of specific intakes of amino acids on bone health; however, mechanistic evidence suggests that a number of amino acids, including five of the nonessential amino acids, may be associated with BMD. In particular, arginine, lysine, alanine, proline, leucine, and glutamine have been shown in vitro to stimulate insulin secretion, which promotes osteoblast growth and differentiation.^15^, ^16^ Arginine has also been shown to stimulate growth hormone secretion thereby promoting production of IGF‐1,^17^ whereas arginine, lysine, and glycine have been associated with an improvement in collagen formation or synthesis.^18^, ^19^ Leucine has a direct effect on the initiation of mRNA translation and is thought to be the most efficient of the branched‐chain amino acids at increasing muscle protein synthesis, which is critical for the maintenance of adequate bone strength and density.^20^

Therefore, we examined for the first time the relationship between intakes of seven specific amino acids with known mechanistic links to bone health, BMD, and prevalence of osteoporosis or osteopenia in a cohort of 3020 healthy women aged 18 to 79 year. Furthermore, we assessed if the dietary source of the amino acids, vegetable or animal, were differentially associated with these outcomes. As genetic factors are strong determinants of BMD, with estimates of heritability in this cohort at 46% to 84%,^21^ we used the discordant monozygotic twin model to examine associations between intake of amino acids and BMD independently of genetic and shared environmental factors. On the basis of previous research, it was hypothesized that participants with higher intakes of amino acids that have been shown in vitro to be associated with bone health (alanine, arginine, glutamic acid, glycine, leucine, lysine, and proline) would be associated with a lower prevalence of osteoporosis and higher BMD.

## Subjects and Methods

### Study population

The current study used data collected from study participants in the TwinsUK registry, a nationwide registry that consists of adult twin volunteers recruited from the general population through national media campaigns in the UK.^22^ All participants were unaware of the specific hypotheses being tested, and were not selected on the basis of the variables being studied. Informed consent was obtained from all participants and ethical approval for the study was gained from St Thomas's Hospital Research Ethics committee. The participants included in this analyses were female, aged 18 to 75 years, and were a sample of the total population group who had completed both food frequency questionnaires (FFQs) and attended for clinical assessment of BMD between 1996 and 2000. Of the 5119 participants who completed an FFQ, 36% (*n* = 1857) were excluded for having an incomplete FFQ (answers for more than 10 food items were left blank) or implausible energy intake (the ratio of energy intake to estimated basal metabolic rate was ≥2 SD above the population mean^23^). A further 2% (*n* = 102) did not attend a clinical session for BMD assessment, resulting in 3160 participants being included in the current analyses. This population has been shown to be representative of the general population in terms of BMD and dietary intake.^22^, ^23^

### Assessment of BMD

Central BMD was measured at the lumbar spine (L_1_ to L_4_) and femoral neck and peripheral BMD at the forearm by dual‐energy X‐ray absorptiometry (DXA) using standard protocols (QDR‐2000W; Hologic, Bedford, MA, USA. Osteoporosis was defined as a *T*‐score of < –2.5 SD below peak female bone mass and osteopenia as a *T*‐score between –1 and –2.49 SD. Following the guidelines of the National Osteoporosis Society these were only defined for postmenopausal women aged over 50 years.^24^

### Assessment of amino acid intakes

Participants completed a 131‐item validated FFQ.^25^, ^26^ Intakes of amino acids were derived predominantly using UK food composition data but with additional data from the U.S. Department of Agriculture.^27^, ^28^ Values for 18 individual amino acids were assigned to each of the foods listed in the FFQ and for we assigned values for each ingredient in the mixed dishes. When values for total protein from the amino acid database and the latest UK food composition tables differed, the amino acid composition of the food items were modified to match the most up‐to‐date data.^29^ Intakes of individual amino acids were calculated as the frequency of each food multiplied by the amino acid content of the food for the appropriate portion size.^30^ All foods were classified as either animal or vegetable origin, and for mixed dishes the proportions contributed from animal and vegetable sources were calculated by breaking down the ingredients into foods that were attributable to a single source.

### Assessment of covariates

Zygosity was ascertained by questionnaire and confirmed via subsequent genotyping as part of genomewide association studies. Intake of energy and nutrients associated with BMD were determined from the FFQ as described in the assessment of amino acid intakes section. Height was measured to the nearest 0.5 cm with the use of a wall‐mounted stadiometer and weight (light clothing only) was measured to the nearest 0.1 kg with digital scales. Information on medication and supplement use, lifestyle, and demographic variables were obtained by standardized nurse‐administered questionnaire. Physical activity was classified as inactive, moderate, and active during work, home, and leisure time using a questionnaire strongly correlated with more in‐depth assessment of time spent in physical activity in this cohort.^31^ The mean time spent in physical activity per week for each physical activity level was as follows: inactive, 16 min; light activity, 36 min; moderate activity, 102 min; and heavy activity, 199 min. Underreporting of energy intake was assessed by calculating the ratio of reported energy intake to estimated energy requirements, based on the Institute of Medicine equations.^32^ A 95% CI for the accuracy of these values was then calculated by taking into account the amount of variation inherent in the methods used to assess energy intake and energy requirements.^33^ Because excluding participants who underreport can introduce considerable bias, underreporting was considered as a covariate in all multivariable models.^34^

### Statistical analysis

Statistical analyses were performed with Stata statistical software version 11.2 (StataCorp, College Station, TX, USA). First, we used the entire sample, treating twins as individuals while accounting for twin pair clustering. Quartiles (Q1 to Q4) of intake were calculated for total protein and the seven amino acids with known mechanistic links to bone health. Analysis of covariance (ANCOVA) was used to calculate adjusted means and evaluate statistical trends in BMD across quartiles and prevalence ratios for low bone mass (osteoporosis and osteopenia combined) were estimated using Poisson regression. There was a significant interaction between amino acid intake and protein source, so all analyses were stratified by source (all, vegetable, animal). All models were adjusted for age (years), current smoking (yes, no), physical activity (inactive, moderately active, active), weight (kg), height (cm), use of hormone replacement therapy (yes, no), use of calcium or vitamin D supplements (yes, no), menopausal status (premenopausal, postmenopausal), underreporting (yes, no), and intakes of alcohol (g), calcium (mg), magnesium (mg), and phosphorous (mg). The intakes of individual amino acids were additionally adjusted for total protein intake (g).

In further analyses we studied monozygotic twin pairs who were discordant for energy‐adjusted intakes of total protein and bone protective amino acids (defined as a within‐pair difference of at least 1 SD) and compared BMD levels in the twins with higher intake versus lower intake using paired sample *t* tests. To eliminate other known environmental influences on BMD we ensured all twin pairs were concordant for menopausal status and there were no significant differences between the higher and lower intake pairs for smoking status, alcohol intake, physical activity, weight, or use of hormone replacement therapy. All analyses were conducted with total protein and amino acids expressed as a percentage of energy in order to best present the data relative to total dietary intake.

## Results

The baseline characteristics of the population of 3160 female participants are presented in Table 1. The average age of participants was 48 years (SD 12.7), 18.5% smoked, and 24% were physically active. Total daily protein intake was 80.5 g (SD 21.6) and contributed 16.5% (SD 2.64) to total energy intake. Of the amino acids under study, glutamic acid (19.8% SD 1.1) and leucine (7.9% SD 0.2) made the greatest contribution to total protein intake, with intakes of 3.2% (SD 0.5) and 1.3% (0.2) of total energy intake, respectively. Of the six amino acids investigated, vegetable sources made the greatest contribution to glutamic acid intake (43% of intake) and the least to intakes of lysine (26% of intake). As expected, all the amino acids were significantly correlated with protein intake, with coefficients ranging from 0.20 for glycine intake to 0.70 for leucine intake (data not shown). Of the postmenopausal participants aged over 50 years (*n* = 1120) 18% were classified as having osteoporosis and 45% as having osteopenia.

**Table 1:** Characteristics and Dietary Intakes of the 3160 Female Participants From the Twins UK Study

		IQR
Characteristics		
Age (years), mean ± SD	48.2 ± 12.7	40.0–58.0
Height (cm), mean ± SD	162 ± 6.12	158.0–166.0
Weight (kg), mean ± SD	66.3 ± 12.0	57.9–72.1
Current smoker (yes), *n* (%)	585 (18.5)	–
Physically active (yes), *n* (%)	758 (24.0)	–
Hormone replacement therapy use (yes), *n* (%)	552 (17.5)	–
Calcium or vitamin D supplement use (yes), *n* (%)	96 (3.0)	–
Menopausal status (postmenopausal), *n* (%)	1509 (47.8)	–
Bone health		
Lumbar spine BMD (g/cm^2^), mean ± SD	0.993 ± 0.14	0.898–1.083
Femoral neck BMD (g/cm^2^), mean ± SD	0.807 ± 0.13	0.716–0.892
Forearm BMD (g/cm^2^), mean ± SD	0.555 ± 0.06	0.523–0.592
Osteoporosis (yes), *n* (%)^a^	202 (18.0)	–
Osteopenia (yes), *n* (%)^a^	502 (44.8)	–
Dietary intake		
Energy (kcal/day), mean ± SD	1980 ± 527	1607–2315
Protein (g/day), mean ± SD	80.5 ± 2.6	14.7–18.0
Alanine (g/day), mean ± SD	3.9 ± 1.1	3.1–4.5
Arginine (g/day), mean ± SD	4.4 ± 1.2	3.6–5.1
Glutamic acid (g/day), mean ± SD	15.9 ± 4.3	12.9–18.7
Glycine (g/day), mean ± SD	3.2 ± 0.9	2.6–3.8
Leucine (g/day), mean ± SD	6.4 ± 1.7	5.2–7.4
Lysine (g/day), mean ± SD	5.4 ± 1.6	4.3–6.4
Proline (g/day), mean ± SD	5.5 ± 1.5	4.5–6.5
Protein, vegetable sources (g/day), mean ± SD	30.6 ± 10.2	23.4–36.0
Alanine, vegetable sources (g/day), mean ± SD	1.3 ± 0.5	1.0–1.6
Arginine, vegetable sources (g/day), mean ± SD	1.8 ± 0.6	1.4–2.1
Glutamic acid, vegetable sources (g/day), mean ± SD	6.8 ± 2.5	5.1–8.3
Glycine, vegetable sources (g/day), mean ± SD	1.3 ± 0.4	1.0–1.5
Leucine, vegetable sources (g/day), mean ± SD	2.2 ± 0.7	1.7–2.6
Lysine, vegetable sources (g/day), mean ± SD	1.4 ± 0.5	1.1–1.7
Proline, vegetable sources (g/day), mean ± SD	2.4 ± 0.9	1.8–2.9
Alcohol (g/day), mean ± SD	9.7 ± 13.5	1.19–13.3
Calcium (mg/day), mean ± SD	1135 ± 374	868–1357
Magnesium (mg/day), mean ± SD	343 ± 92.7	278–398
Phosphorous (mg/day), mean ± SD	1521 ± 409	1224–1784

Values are mean ± SD or *n* (%); *n* = 3160.

^a^ Postmenopausal participants aged over 50 years; *n* = 1120.

As shown in Table 2, in multivariable analyses a higher intake of total protein was significantly associated with a higher BMD at the spine (Q4 to Q1: 0.017 g/cm^2^, SE 0.01, *p* trend 0.035), and forearm (Q4 to Q1: 0.010 g/cm^2^, SE 0.003, *p* trend 0.002), with no associations observed for BMD at the femoral neck (*p* trend = 0.83) or with prevalence of osteoporosis or osteopenia (Table 3, *p* trend = 0.75) Higher intake of vegetable protein was not associated with BMD at any site, although the prevalence (PR) of osteoporosis or osteopenia was 13% lower in Q4 of vegetable protein intake compared to Q1 (PR 0.87; 95% CI, 0.8 to 1.0; *p* trend 0.029). No associations with BMD or prevalence of osteoporosis or osteopenia were observed for animal protein.

**Table 2:** Associations Between Intakes of Total Protein, Bone‐Protective Amino Acids and Bone Mineral Density (g/cm2), by Food Source, in 3160 Females Aged 18 to 75 Years

	Protein	Alanine	Arginine	Glutamic acid	Glycine	Leucine	Lysine	Proline
All								
Spine BMD (g/cm^2^)								
Q1	0.984 ± 0.01	0.982 ± 0.01	0.984 ± 0.01	0.985 ± 0.01	0.984 ± 0.01	0.978 ± 0.01	0.981 ± 0.01	0.985 ± 0.01
Q2	0.992 ± 0.01	0.991 ± 0.01	0.990 ± 0.01	0.989 ± 0.01	0.996 ± 0.01	0.995 ± 0.01	0.991 ± 0.01	0.989 ± 0.01
Q3	0.995 ± 0.01	0.997 ± 0.01	0.995 ± 0.01	0.994 ± 0.01	0.991 ± 0.01	0.995 ± 0.01	0.995 ± 0.01	0.993 ± 0.01
Q4	1.000 ± 0.01	1.002 ± 0.01	1.003 ± 0.01	1.004 ± 0.01	1.000 ± 0.01	1.003 ± 0.01	1.004 ± 0.01	1.004 ± 0.01
*p* trend	0.035	0.017	0.015	0.018	0.097	0.006	0.008	0.018
Femoral neck BMD (g/cm^2^)								
Q1	0.806 ± 0.004	0.807 ± 0.005	0.806 ± 0.004	0.809 ± 0.004	0.808 ± 0.005	0.804 ± 0.004	0.805 ± 0.004	0.807 ± 0.004
Q2	0.811 ± 0.004	0.810 ± 0.004	0.811 ± 0.004	0.806 ± 0.004	0.813 ± 0.004	0.810 ± 0.004	0.811 ± 0.004	0.805 ± 0.004
Q3	0.802 ± 0.004	0.803 ± 0.004	0.802 ± 0.004	0.804 ± 0.004	0.801 ± 0.004	0.803 ± 0.004	0.804 ± 0.004	0.807 ± 0.004
Q4	0.810 ± 0.005	0.810 ± 0.005	0.811 ± 0.004	0.811 ± 0.005	0.807 ± 0.005	0.812 ± 0.005	0.810 ± 0.005	0.810 ± 0.005
*p* trend	0.831	0.764	0.603	0.766	0.605	0.365	0.659	0.594
Forearm BMD (g/cm^2^)								
Q1	0.550 ± 0.002	0.550 ± 0.002	0.551 ± 0.002	0.551 ± 0.002	0.551 ± 0.002	0.548 ± 0.002	0.549 ± 0.002	0.551 ± 0.002
Q2	0.555 ± 0.002	0.556 ± 0.002	0.555 ± 0.002	0.555 ± 0.002	0.556 ± 0.002	0.557 ± 0.002	0.557 ± 0.002	0.555 ± 0.002
Q3	0.555 ± 0.002	0.554 ± 0.002	0.554 ± 0.002	0.555 ± 0.002	0.553 ± 0.002	0.555 ± 0.002	0.555 ± 0.002	0.556 ± 0.002
Q4	0.560 ± 0.002	0.559 ± 0.002	0.560 ± 0.002	0.560 ± 0.002	0.560 ± 0.002	0.560 ± 0.002	0.560 ± 0.002	0.558 ± 0.002
*p* trend	0.002	0.017	0.007	0.013	0.039	0.001	0.003	0.031
Intake (%kcal)^a^								
Q1	13.3 ± 1.15	0.61 ± 0.07	0.70 ± 0.07	2.68 ± 0.21	0.51 ± 0.05	1.04 ± 0.09	0.83 ± 0.10	0.93 ± 0.08
Q2	15.5 ± 0.46	0.73 ± 0.03	0.84 ± 0.03	3.09 ± 0.08	0.61 ± 0.02	1.23 ± 0.04	1.03 ± 0.04	1.07 ± 0.03
Q3	17.1 ± 0.50	0.83 ± 0.03	0.94 ± 0.03	3.36 ± 0.09	0.69 ± 0.02	1.36 ± 0.04	1.17 ± 0.05	1.17 ± 0.03
Q4	19.9 ± 1.65	0.99 ± 0.10	1.12 ± 0.11	3.84 ± 0.28	0.83 ± 0.08	1.59 ± 0.14	1.44 ± 0.15	1.34 ± 0.10
Vegetable								
Spine BMD (g/cm^2^)								
Q1	0.989 ± 0.01	0.989 ± 0.01	0.988 ± 0.01	0.988 ± 0.01	0.987 ± 0.01	0.983 ± 0.01	0.988 ± 0.01	0.992 ± 0.01
Q2	0.986 ± 0.01	0.995 ± 0.01	0.995 ± 0.01	0.984 ± 0.01	0.992 ± 0.01	0.987 ± 0.01	0.993 ± 0.01	0.990 ± 0.01
Q3	0.999 ± 0.01	0.988 ± 0.01	0.993 ± 0.01	1.002 ± 0.01	0.997 ± 0.01	1.000 ± 0.01	0.990 ± 0.01	0.999 ± 0.01
Q4	0.998 ± 0.01	0.999 ± 0.01	0.995 ± 0.01	0.997 ± 0.01	0.995 ± 0.01	1.000 ± 0.01	1.001 ± 0.01	0.990 ± 0.01
*p* trend	0.265	0.361	0.541	0.205	0.359	0.050	0.150	0.975
Femoral neck BMD (g/cm^2^)								
Q1	0.811 ± 0.004	0.810 ± 0.005	0.810 ± 0.005	0.806 ± 0.005	0.808 ± 0.005	0.809 ± 0.005	0.809 ± 0.005	0.807 ± 0.005
Q2	0.808 ± 0.004	0.811 ± 0.004	0.808 ± 0.004	0.805 ± 0.004	0.812 ± 0.004	0.803 ± 0.004	0.807 ± 0.004	0.808 ± 0.004
Q3	0.808 ± 0.004	0.802 ± 0.004	0.806 ± 0.004	0.812 ± 0.004	0.807 ± 0.004	0.813 ± 0.004	0.802 ± 0.004	0.811 ± 0.004
Q4	0.803 ± 0.005	0.807 ± 0.005	0.806 ± 0.005	0.807 ± 0.005	0.803 ± 0.005	0.805 ± 0.005	0.811 ± 0.005	0.804 ± 0.005
*p* trend	0.347	0.592	0.625	0.781	0.436	0.789	0.875	0.630
Forearm BMD (g/cm^2^)								
Q1	0.553 ± 0.002	0.553 ± 0.002	0.552 ± 0.002	0.554 ± 0.002	0.552 ± 0.002	0.553 ± 0.002	0.553 ± 0.002	0.556 ± 0.002
Q2	0.557 ± 0.002	0.557 ± 0.002	0.556 ± 0.002	0.557 ± 0.002	0.558 ± 0.002	0.554 ± 0.002	0.556 ± 0.002	0.557 ± 0.002
Q3	0.558 ± 0.002	0.556 ± 0.002	0.557 ± 0.002	0.555 ± 0.002	0.557 ± 0.002	0.559 ± 0.002	0.556 ± 0.002	0.557 ± 0.002
Q4	0.553 ± 0.002	0.555 ± 0.002	0.556 ± 0.002	0.554 ± 0.002	0.554 ± 0.002	0.555 ± 0.002	0.556 ± 0.002	0.550 ± 0.002
*p* trend	0.848	0.694	0.293	0.818	0.633	0.578	0.533	0.082
Intake (%kcal)^a^								
Q1	4.7 ± 0.47	0.21 ± 0.02	0.27 ± 0.03	1.02 ± 0.12	0.19 ± 0.02	0.34 ± 0.03	0.21 ± 0.02	0.35 ± 0.04
Q2	5.7 ± 0.21	0.25 ± 0.01	0.33 ± 0.01	1.25 ± 0.06	0.24 ± 0.01	0.41 ± 0.02	0.26 ± 0.01	0.43 ± 0.02
Q3	6.4 ± 0.24	0.28 ± 0.01	0.38 ± 0.02	1.44 ± 0.06	0.27 ± 0.01	0.47 ± 0.02	0.30 ± 0.01	0.50 ± 0.02
Q4	7.9 ± 1.04	0.36 ± 0.05	0.49 ± 0.09	1.80 ± 0.23	0.34 ± 0.05	0.57 ± 0.08	0.38 ± 0.07	0.63 ± 0.08
Animal								
Spine BMD (g/cm^2^)								
Q1	0.983 ± 0.01	0.987 ± 0.01	0.984 ± 0.01	0.984 ± 0.01	0.990 ± 0.01	0.989 ± 0.01	0.981 ± 0.01	0.985 ± 0.01
Q2	0.992 ± 0.01	0.992 ± 0.01	0.993 ± 0.01	0.997 ± 0.01	0.996 ± 0.01	0.992 ± 0.01	0.991 ± 0.01	0.997 ± 0.01
Q3	0.994 ± 0.01	0.992 ± 0.01	0.993 ± 0.01	0.989 ± 0.01	0.989 ± 0.01	0.992 ± 0.01	0.994 ± 0.01	0.989 ± 0.01
Q4	1.003 ± 0.01	1.000 ± 0.01	1.001 ± 0.01	1.001 ± 0.01	0.997 ± 0.01	0.999 ± 0.01	1.005 ± 0.01	1.000 ± 0.01
*p* trend	0.108	0.112	0.297	0.244	0.654	0.391	0.020	0.290
Femoral neck BMD (g/cm^2^)								
Q1	0.808 ± 0.006	0.809 ± 0.004	0.806 ± 0.005	0.807 ± 0.006	0.810 ± 0.006	0.812 ± 0.006	0.806 ± 0.005	0.807 ± 0.005
Q2	0.810 ± 0.004	0.810 ± 0.004	0.811 ± 0.004	0.812 ± 0.004	0.809 ± 0.004	0.810 ± 0.004	0.809 ± 0.004	0.810 ± 0.004
Q3	0.808 ± 0.004	0.805 ± 0.004	0.805 ± 0.004	0.805 ± 0.004	0.805 ± 0.004	0.805 ± 0.004	0.806 ± 0.004	0.808 ± 0.004
Q4	0.805 ± 0.006	0.805 ± 0.006	0.807 ± 0.005	0.806 ± 0.006	0.805 ± 0.006	0.803 ± 0.006	0.809 ± 0.005	0.806 ± 0.006
*p* trend	0.628	0.783	0.676	0.733	0.539	0.284	0.883	0.795
Forearm BMD (g/cm^2^)								
Q1	0.552 ± 0.003	0.552 ± 0.003	0.550 ± 0.003	0.552 ± 0.003	0.551 ± 0.003	0.554 ± 0.003	0.550 ± 0.003	0.555 ± 0.003
Q2	0.557 ± 0.002	0.557 ± 0.002	0.556 ± 0.002	0.557 ± 0.002	0.558 ± 0.002	0.557 ± 0.002	0.556 ± 0.002	0.556 ± 0.002
Q3	0.555 ± 0.002	0.555 ± 0.002	0.555 ± 0.002	0.555 ± 0.002	0.555 ± 0.002	0.554 ± 0.002	0.556 ± 0.002	0.557 ± 0.002
Q4	0.556 ± 0.003	0.556 ± 0.003	0.558 ± 0.002	0.556 ± 0.003	0.556 ± 0.003	0.556 ± 0.003	0.558 ± 0.002	0.553 ± 0.003
*p* trend	0.647	0.102	0.463	0.635	0.501	0.922	0.113	0.656
Intake (%kcal)^a^								
Q1	6.8 ± 1.35	0.32 ± 0.07	0.33 ± 0.08	1.25 ± 0.24	0.24 ± 0.06	0.57 ± 0.11	0.53 ± 0.11	0.44 ± 0.08
Q2	9.3 ± 0.50	0.46 ± 0.03	0.47 ± 0.03	1.69 ± 0.09	0.35 ± 0.02	0.78 ± 0.04	0.74 ± 0.04	0.59 ± 0.03
Q3	11.0 ± 0.52	0.56 ± 0.03	0.57 ± 0.03	1.99 ± 0.09	0.43 ± 0.03	0.92 ± 0.04	0.89 ± 0.04	0.70 ± 0.03
Q4	14.0 ± 1.71	0.73 ± 0.10	0.75 ± 0.10	2.51 ± 0.29	0.57 ± 0.08	1.16 ± 0.14	1.17 ± 0.14	0.88 ± 0.10

Values are mean ± SE adjusted for age, current smoking, physical activity, weight, height, use of hormone replacement therapy, use of calcium and vitamin D supplements, menopausal status, underreporting, and intakes of alcohol, calcium, magnesium, and phosphorous. The intakes of individual amino acids were additionally adjusted for absolute intakes of the other amino acids (g) and intake of total protein and amino acids from vegetable and animal sources were mutually adjusted for one another. *p* trend was calculated using ANCOVA.

Q1–Q4 = quartiles 1 to 4; ANCOVA = analyses of covariance.

^a^ Unadjusted mean ± SD intakes.

**Table 3:** Prevalence Ratios of Osteoporosis or Osteopenia by Quintile of Total Protein and Bone‐Protective Amino Acids, by Food Source, in 1120 Postmenopausal Females Aged Over 50 years

	Protein	Alanine	Arginine	Glutamic acid	Glycine	Leucine	Lysine	Proline
All								
Q1	1.00 (ref)	1.00 (ref)	1.00 (ref)	1.00 (ref)	1.00 (ref)	1.00 (ref)	1.00 (ref)	1.0 (ref)
Q2	1.01 (0.9–1.1)	0.98 (0.9–1.1)	0.99 (0.9–1.1)	1.00 (0.9–1.1)	0.97 (0.9–1.1)	0.98 (0.9–1.1)	1.00 (0.9–1.1)	1.03 (0.9–1.2)
Q3	1.15 (1.0–1.3)	1.04 (0.9–1.2)	1.01 (0.9–1.1)	1.01 (0.9–1.2)	1.04 (0.9–1.2)	1.06 (0.9–1.2)	1.04 (0.9–1.2)	1.00 (0.9–1.1)
Q4	1.00 (0.9–1.1)	0.94 (0.8–1.1)	0.93 (0.8–1.1)	0.94 (0.8–1.1)	0.96 (0.8–1.1)	0.97 (0.8–1.1)	0.99 (0.9–1.1)	0.95 (0.8–1.1)
*p* trend	0.747	0.468	0.362	0.418	0.720	0.795	0.973	0.395
Vegetable								
Q1	1.00 (ref)	1.00 (ref)	1.00 (ref)	1.00 (ref)	1.00 (ref)	1.00 (ref)	1.00 (ref)	1.00 (ref)
Q2	0.92 (0.8–1.0)	0.89 (0.8–1.0)	0.88 (0.8–1.0)	0.92 (0.8–1.0)	0.94 (0.8–1.0)	0.92 (0.8–1.0)	0.89 (0.8–1.0)	0.92 (0.8–1.0)
Q3	0.92 (0.8–1.0)	0.89 (0.8–1.0)	0.89 (0.8–1.0)	0.94 (0.8–1.1)	0.87 (0.8–1.0)	0.91 (0.8–1.0)	0.92 (0.8–1.0)	0.94 (0.8–1.0)
Q4	0.87 (0.8–1.0)	0.86 (0.8–1.0)	0.81 (0.7–0.9)	0.92 (0.8–1.0)	0.86 (0.8–1.0)	0.87 (0.8–1.0)	0.85 (0.8–1.0)	0.92 (0.8–1.0)
*p* trend	0.029	0.027	0.001	0.241	0.009	0.026	0.013	0.283
Animal								
Q1	1.00 (ref)	1.00 (ref)	1.00 (ref)	1.00 (ref)	1.00 (ref)	1.00 (ref)	1.00 (ref)	1.00 (ref)
Q2	1.07 (1.0–1.2)	1.06 (0.9–1.2)	1.03 (0.9–1.2)	1.02 (0.9–1.1)	1.10 (1.0–1.2)	1.05 (0.9–1.2)	1.08 (1.0–1.2)	1.02 (0.9–1.1)
Q3	1.05 (0.9–1.2)	1.09 (1.0–1.2)	1.08 (1.0–1.2)	1.07 (1.0–1.2)	1.11 (1.0–1.2)	1.08 (1.0–1.2)	1.05 (0.9–1.2)	1.04 (0.9–1.2)
Q4	1.06 (0.9–1.2)	1.09 (1.0–1.2)	1.06 (0.9–1.2)	1.01 (0.9–1.2)	1.09 (1.0–1.2)	1.06 (0.9–1.2)	1.11 (1.0–1.2)	1.01 (0.9–1.2)
*p* trend	0.471	0.177	0.277	0.709	0.211	0.354	0.152	0.921

Values are the adjusted prevalence ratios (95% CI) for low bone mass (osteoporosis and osteopenia combined). Low bone mass was defined as a *T*‐score less than –1 at the spine, hip or forearm. Prevalence ratios were adjusted for age, current smoking, physical activity, weight, height, use of hormone replacement therapy, use of calcium and vitamin D supplements, underreporting, and intakes of alcohol, calcium, magnesium, and phosphorous. The intakes of individual amino acids were additionally adjusted for absolute intakes of the other amino acids and intake of total protein and amino acids from vegetable and animal sources were mutually adjusted for one another. *p* trend was calculated using Poisson regression with robust variance.

Q1–Q4 = quartiles 1 to 4.

Higher intakes of six of the bone‐protective amino acids were significantly associated with higher BMD at the spine and forearm (only glycine intake was not associated; Table 2). At the spine the magnitudes of association ranged from 0.019 g/cm^2^ (SE 0.008, *p* trend 0.018) for proline to 0.025 g/cm^2^ (SE 0.008, *p* trend 0.018) for leucine, when comparing extreme quartiles of intake. For forearm BMD, a difference of 0.009 g/cm^2^ was observed between extreme quartiles of alanine (SE 0.003, *p* trend 0.017), arginine (SE 0.003, *p* trend 0.007), and glutamic acid (SE 0.003; *p* trend 0.013) intake. The remaining three amino acids, leucine, lysine, and proline, were associated with 0.012 g/cm^2^ (SE 0.003, *p* trend 0.001), 0.011 g/cm^2^ (SE 0.003, *p* trend 0.003), and 0.007 g/cm^2^ (SE 0.003, *p* trend 0.031) differences in forearm BMD, respectively, comparing extreme quartiles of intake.

There were no notable associations between intakes of these seven amino acids and BMD when stratified by vegetable or animal sources. However, the prevalence of osteoporosis or osteopenia was significantly lower in the highest compared to the lowest quartile of amino acid intake from vegetable sources for five of the seven bone‐protective amino acids including alanine, arginine, glycine, leucine, and lysine. The results indicated a 13% to 19% lower prevalence of low bone mass in the highest compared to the lowest quintile of vegetable intake for alanine (PR 0.86; 95% CI, 0.8 to 1.0; *p* trend 0.027), arginine (PR 0.81; 95% CI, 0.7 to 0.9; *p* trend 0.001), glycine (PR 0.86; 95% CI, 0.8 to 1.0; *p* trend 0.009), leucine (PR 0.87; 95% CI, 0.8 to 1.0; *p* trend 0.026), and lysine (PR 0.85; 95% CI, 0.8 to 1.0; *p* trend 0.013).

**Figure 1:**
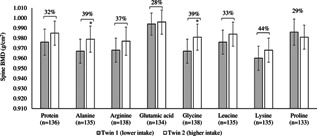
Spine BMD in monozygotic twins discordant for intakes of total protein and bone‐protective amino acids. Discordance was defined as a within‐pair difference in intake of at least 1 SD. Data are means ± SE of spine BMD in twin 1 with lower intake and twin 2 with higher intake. The data values on the graph represent the percentage difference in intake between the lower‐intake and higher‐ intake twin. *p < 0.05 calculated using paired sample *t* tests comparing higher‐intake and lower‐intake twin‐pairs.

In our co‐twin case control analyses we examined monozygotic twin pairs discordant for protein and amino acid intake and concordant for menopausal status. Intakes of total protein differed by 32% (14.6% energy [SD 2.3] versus 19.3% energy [SD 2.7]) between the higher‐intake and lower‐intake twins and intakes of the amino acids differed by 28% for glutamic acid (2.9% energy [SD 0.4] versus 3.7% energy [SD 0.5]) to 44% for lysine (1.0% energy [SD 0.2] versus 1.4% energy [SD 0.3]). Compared to the twins with lower intakes, twins with higher intakes of alanine and glycine had significantly higher BMD at the spine than their co‐twins (Fig. 1). Specifically, there were differences in BMD of 0.012 g/cm^2^ (SE 0.01; *p* = 0.039) for alanine and 0.014 g/cm^2^ (SE 0.01; *p* = 0.026) for glycine. There were no significant differences in spine BMD between the higher‐intake and lower‐intake twins for total protein (0.009 g/cm^2^; SE 0.01; *p* = 0.07), arginine (0.009 g/cm^2^; SE 0.01; *p* = 0.11), glutamic acid (0.002 g/cm^2^; SE 0.01; *p* = 0.38), leucine (0.009 g/cm^2^; SE 0.01; *p* = 0.07), lysine (0.009 g/cm^2^; SE 0.01; *p* = 0.07), or proline (0.005 g/cm^2^; SE 0.01; *p* = 0.73).

Sensitivity analyses in our cross‐sectional analyses replacing intake of the individual amino acids with intake of total protein as a covariate did not markedly change our results. Furthermore, substituting menopausal status with years since menopause did not change the direction or magnitude of the associations reported, although significance was lost for the association between total protein intake and spine BMD (Q5 to Q1: 0.02; SE 0.01; *p* trend = 0.06) and proline intake and forearm BMD (Q5 to Q1: 0.01; SE 0.003; *p* trend 0.07).

## Discussion

To our knowledge, this is the first cross‐sectional study to examine associations between dietary intakes of amino acids, which have previously been linked in mechanistic research to bone health, BMD, and prevalence of osteoporosis or osteopenia. The twin population provided the unique opportunity to control for potentially confounding genetic influences by comparing identical twins who were discordant for protein or amino acid intake. The monozygotic twins with higher intakes of alanine and glycine had significantly higher BMD at the spine than their co‐twins. Specifically, there were differences in BMD of 0.012 g/cm^2^ (SE 0.01; *p* = 0.039) for alanine and 0.014 g/cm^2^ (SE 0.01; *p* = 0.026) for glycine. For comparison, the magnitude of these associations equated to the coefficients for spine BMD we observed in our multivariable adjusted models for a 5‐year increase in age (–0.011 g/cm^2^), smoking versus not smoking (–0.008 g/cm^2^), and physical inactivity versus activity (–0.003 g/cm^2^).

Our novel data also show in this population of middle aged females that as well as a protective effect for total protein intake, higher intake of six bone‐protective amino acids (alanine, arginine, glutamic acid, leucine, lysine, and proline) were significantly associated with higher BMD at the spine and forearm. These associations were independent of established lifestyle factors and medications that are known to be related to bone health as well as other dietary factors including alcohol, calcium, phosphorous, and magnesium intakes.

Women in the highest quartile of protein intake (19.9% energy) had significantly higher BMD in the spine (0.017 g/cm^2^) and forearm (0.010 g/cm^2^) compared to those in the lowest quartile (13.3% energy). This result confirms previous cross‐sectional and prospective findings of a positive relationship between higher total protein intake and higher BMD in women.^6^, ^7^, ^8^, ^9^ We found no association between intake of vegetable protein and BMD, contradicting some previous studies,^10^, ^11^, ^12^ but we did observe a clear association between intake of vegetable protein and prevalence of low bone mass (osteoporosis or osteopenia). The prevalence of osteoporosis or osteopenia for postmenopausal women in the highest quartile of vegetable protein intake was 67.2%, compared to 73.2% in the lowest quartile. Vegetable food sources are known to provide base rather than acid precursors and an acid‐forming diet has been shown to increase urinary calcium excretion, stimulate osteoclast action, and inhibit osteoblastic action.^35^, ^36^ We acknowledge, however, that cereal products, which made a significant contribution to intakes of amino acids from vegetable sources in this cohort, are associated with a higher acid‐base load.^37^ It was not possible, in this exploratory analyses, to understand the mechanisms for these opposing findings for BMD and prevalence of low bone mass, and therefore it will be important for future studies to estimate both parameters when investigating relationships with amino acid intakes.

Findings for the individual amino acids we investigated were of a similar magnitude to those for total protein and are consistent with mechanistic research that links intake of specific amino acids to parameters of bone health. For example, in animal models a low protein diet induced a decrease in BMD and subsequent administration of dietary essential amino acid supplements, in the same relative proportions as casein, were found to cause an increase bone strength as a result of increased urinary deoxypyridinoline excretion, reflecting increased bone resorption, and plasma IGF‐1 levels.^38^ Furthermore, supplementation with L‐arginine (2 g/day) for 2 years increased BMD by 11.6% in 150 osteoporotic postmenopausal women^39^; this dose reflected 45% of mean arginine intakes in the current study. Interestingly, in comparison to branched chain amino acids, aromatic amino acids, which were not investigated in the current research, were also found to increase IGF‐1 and calcium absorption.^40^

The magnitude of the associations we observed ranged from 0.019 g/cm^2^ to 0.024 g/cm^2^ (2.4% of mean BMD) at the spine and 0.009 g/cm^2^ to 0.012 g/cm^2^ (2.2% of mean BMD) at the forearm. The size of these associations corresponds to those previously reported for a 100‐mg increase in magnesium (0.020 g/cm^2^; 2% of mean BMD) or vitamin C (0.017 g/cm^2^) and supplementation with calcium (1.66% increase in mean BMD for lumbar spine; 1.64% increase in mean BMD for hip).^41^, ^42^, ^43^ The greatest difference in BMD of 0.024 g/cm^2^ was observed between extreme quintiles of leucine intake; this corresponded to a difference in intake of 0.6% energy or 1.4 g. This intake could readily be incorporated in the diet through animal sources, eg, one salmon steak (80 g) contains 1.6 g leucine, or through a variety of vegetable sources, eg, one medium avocado (145 g = 0.2 g leucine), or an average portion of brown rice (180 g = 0.3 g leucine), almonds (40 g = 0.5 g leucine), or high fiber breakfast cereal (40 g = 0.3 g leucine). These findings show that the associations we observed in the current study are related to dietary achievable intakes of amino acids; however, further research is required before recommendations on amino acid intakes in relation to bone health can be made.

It has been proposed that the relationship between increased dietary protein and bone health is strengthened in the presence of calcium sufficiency and adequate intakes of potassium and fruit and vegetables, which are alkalinogenic and therefore decrease urinary calcium excretion.^44^ In terms of calcium intake, previous data from the Framingham cohort have shown that calcium intake modifies the association between protein intake and the risk of hip fracture, and a ratio greater than 20 g calcium per 1 g protein has been suggested as sufficient for bone protection.^12^, ^45^, ^46^ In the current study less than 5% of the cohort were found to have a ratio of calcium to protein at or above 20 (mean 14.0, SD 3.1; data not shown) and it is therefore reasonable to suggest that our findings may have been strengthened with higher calcium intakes. It was also notable that these findings were observed in a cohort of women with a wide age range and who reported high levels of physical activity because we would expect changes in BMD to be less pronounced in this group. There were inconsistencies in our findings at different BMD sites with no associations observed at the femoral neck. The femoral neck contains high levels of cortical bone, as compared to the spine and forearm, which are higher in trabecular bone. Trabecular bone may be more sensitive to dietary changes than cortical bone, and dietary protein intake has been shown to be positively correlated with trabecular but not cortical bone status.^47^

Strengths of the current study include the large sample of well‐characterized participants, objective assessment of bone density by DXA, the gold standard for measurement of BMD, and by specifically studying monozygotic twins, the examination of associations independent of genetic confounding. Our findings relate to women of a wide age range, although our analyses of osteoporosis prevalence in postmenopausal women also showed associations among women who had reached peak bone mass. It was notable that the associations observed in the current analyses were in a population with only 18% classified as osteoporotic and it is plausible that these associations would be more pronounced in a cohort containing a higher proportion of at risk participants. These participants have been shown to be representative of the general population in terms of BMD and diet,^22^, ^23^ and these data have been used in previous studies of dietary exposure and BMD.^48^ The FFQ used in the current study was previously validated against 24‐hour recalls and was shown to classify over 85% of participants into the same or adjacent quintile of protein intake, demonstrating its ability to rank participants according to their habitual protein intake.^49^ Furthermore, the FFQ has been shown to be a valid tool to rank participants according to high and low intakes of amino acids, and the correlations with intakes from weighted dietary records are in line with those for other macronutrients.^50^

The limitations of the current study include the cross‐sectional design, which meant we were unable to infer causality, and we cannot exclude the possibility of residual confounding. However, given the detailed adjustment for a range of dietary and lifestyle confounder variables (such as age, smoking, physical activity, BMI, supplement use, and intake of other nutrients associated with bone health including alcohol, calcium, magnesium, and phosphorous) it is unlikely that these would account for the observed results. Validated biomarkers, such as 24‐hour urine nitrogen, are available for total protein intake but they were not measured in the current study and may have reduced potential measurement error.^51^ Finally, our results relate only to women and because of the differences in rates of bone loss between men and women these findings cannot be extrapolated to other population groups.

In conclusion, these novel data suggest a beneficial role for selected amino acids on BMD and prevalence of low bone mass, with significant associations observed for BMD similar in magnitude to those previously reported for other nutrients including magnesium, calcium, and vitamin C.^41^, ^42^, ^43^ Furthermore, consuming a higher proportion of amino acids from vegetable sources than from animal sources was beneficial in terms of the prevalence of osteoporosis or osteopenia, and intakes of the amino acids associated with the current findings could be incorporated into the diet from readily available vegetable sources.

## Disclosures

All authors state that they have no conflicts of interest.
